# *In silico* analyses of diversity and dissemination of antimicrobial resistance genes and mobile genetics elements, for plasmids of enteric pathogens

**DOI:** 10.3389/fmicb.2022.1095128

**Published:** 2023-01-26

**Authors:** Suad Algarni, Jing Han, Dereje D. Gudeta, Bijay K. Khajanchi, Steven C. Ricke, Young Min Kwon, Douglas D. Rhoads, Steven L. Foley

**Affiliations:** ^1^Division of Microbiology, National Center for Toxicological Research, Food and Drug Administration, Jefferson, AR, United States; ^2^Cell and Molecular Biology Program, University of Arkansas, Fayetteville, AR, United States; ^3^Meat Science & Animal Biologics Discovery Program and Department of Animal and Dairy Sciences, University of Wisconsin, Madison, WI, United States

**Keywords:** plasmids, antimicrobial resistance, disinfectant resistance, metal resistance, mobile genetic elements, Enterobacteriaceae

## Abstract

**Introduction:**

The antimicrobial resistance (AMR) mobilome plays a key role in the dissemination of resistance genes encoded by mobile genetics elements (MGEs) including plasmids, transposons (Tns), and insertion sequences (ISs). These MGEs contribute to the dissemination of multidrug resistance (MDR) in enteric bacterial pathogens which have been considered as a global public health risk.

**Methods:**

To further understand the diversity and distribution of AMR genes and MGEs across different plasmid types, we utilized multiple sequence-based computational approaches to evaluate AMR-associated plasmid genetics. A collection of 1,309 complete plasmid sequences from Gammaproteobacterial species, including 100 plasmids from each of the following 14 incompatibility (Inc) types: A/C, BO, FIA, FIB, FIC, FIIA, HI1, HI2, I1, K, M, N, P except W, where only 9 sequences were available, was extracted from the National Center for Biotechnology Information (NCBI) GenBank database using BLAST tools. The extracted FASTA files were analyzed using the AMRFinderPlus web-based tools to detect antimicrobial, disinfectant, biocide, and heavy metal resistance genes and ISFinder to identify IS/Tn MGEs within the plasmid sequences.

**Results and Discussion:**

*In silico* prediction based on plasmid replicon types showed that the resistance genes were diverse among plasmids, yet multiple genes were widely distributed across the plasmids from enteric bacterial species. These findings provide insights into the diversity of resistance genes and that MGEs mediate potential transmission of these genes across multiple plasmid replicon types. This notion was supported by the observation that many IS/Tn MGEs and resistance genes known to be associated with them were common across multiple different plasmid types. Our results provide critical insights about how the diverse population of resistance genes that are carried by the different plasmid types can allow for the dissemination of AMR across enteric bacteria. The results also highlight the value of computational-based approaches and *in silico* analyses for the assessment of AMR and MGEs, which are important elements of molecular epidemiology and public health outcomes.

## Introduction

Technological and bioinformatics advancements in *in silico* lab-based tools now allow for the replacement of several of the traditional microbiology laboratory methods, potentially increasing analytical throughput and reducing costs. The value of this replacement was particularly evident during the coronavirus-19 (COVID-19) pandemic, when in-person staffing numbers were reduced to maximize physical distancing and remote work became more common. Whole genome sequencing (WGS) is a key approach that provides comprehensive data assessment on the genetics of bacterial pathogens and can potentially replace laboratory-intensive methods. Indeed, several tools and approaches have already been developed to identify the serotype of the isolates, resistance genotypes, plasmid replicon sequence and putative virulence gene content using WGS data (Carattoli et al., 2014; Mao et al., 2015; Zhang et al., 2015). The online bioinformatics tools PlasmidFinder and ResFinder developed by the Center for Genomic Epidemiology, and AMRFinderPlus available through the National Center for Biotechnology Information (NCBI), analyze DNA sequences to identify the resistance genes and factors that can contribute to the transmission of resistance (Zankari et al., 2012; Tyson et al., 2015; Feldgarden et al., 2019). These are important services for antimicrobial resistance (AMR) diagnostics, epidemiological surveillance, and outbreak investigations.

The mobilome encompasses mobile genetic elements (MGEs), including transposons (Tns), insertion sequences (ISs), gene cassettes, integrons, resistance islands, integrative and conjugative elements (ICEs) and plasmids, that can contribute to the spread of genes in a microbial population (Algarni et al., 2022). ISFinder is a bioinformatics tool used for identifying and classifying IS elements and Tn elements which usually carry accessory genes encoding different resistance and/or virulence functions (Siguier et al., 2006; Algarni et al., 2022). One of the major challenges of utilizing WGS data to predict bacterial functions has been the computational horsepower needed to analyze large scale datasets and the bioinformatics expertise to execute some of the analyses programs to achieve the analyses (Tyson et al., 2015). To help overcome some of these limitations, investigators have worked to develop more user-friendly interfaces and to provide resources that allow for analyses to be done remotely utilizing core computing capacity. This study takes advantage of some of these *in silico* tools to identify the plasmid replicon sequences, predict AMR content, and MGEs in enteric bacterial species, including *Salmonella enterica, Escherichia coli, Klebsiella pneumoniae* and *Shigella* spp. The rapid implementation of WGS for epidemiological investigations and the development of improved plasmid sequencing approaches has made more data available to further understand AMR epidemiology, identify the mechanisms that lead to development of AMR phenotypes and improve the detection of resistant strains (Zankari et al., 2012).

These molecular epidemiology efforts aid in understanding the transmission of antimicrobial resistant organisms between humans and other animal species, which can escalate AMR spread across species (e.g., among household pets and owners) or through the food supply (Blondeau, 2017). Multidrug resistance (MDR) has been increasing in prevalence as shown in the many studies examining the relationship between enteric bacterial species and the prevalence of antimicrobial, disinfectant, biocide, and heavy metal resistance encoded on plasmids (Johnson et al., 2007; Deng et al., 2018; McCarlie et al., 2020). Disinfectants, biocides and metal ions are extremely important for the control of infection and/or microbial contamination in the environment. The investigation of heavy metal resistance (HMR) genes in different environments, specifically in *S. enterica* and *E. coli*, suggests that they are often mediated by plasmids. Multiple studies have shown that disinfectant/biocide resistance (DBR), HMR and AMR genes can be co-located on MGEs (Johnson et al., 2007; Han et al., 2012; Deng et al., 2018).

Plasmids are well known as key components of bacterial strains that display high levels of AMR and provide a source for the dissemination of resistance genes (Mathers et al., 2015). Most emerging multidrug-resistant strains rely on a variety of plasmids that can be classified into various incompatibility (Inc) groups, with some of the key ones for Enterobacteriaceae include IncA/C, B/O, FIA, FIB, FIC, FIIA, HI1, HI2, I1, L/M, N, P and W which are associated with resistance to clinically important antibiotics (Carattoli, 2009; Rozwandowicz et al., 2018). In addition to Inc. grouping, some other classification schemes for plasmids that rely on different sequences have been developed, such as those associated with mobilization, which aims to define different plasmid groups associated with conjugation experiments (Petersen, 2011; Rozwandowicz et al., 2018; McMillan et al., 2020). Although the extensive plasmid Inc. typing scheme has already been used to investigate the dissemination of antimicrobial resistant pathogens, further efforts are still required to facilitate epidemiological tracking for identification and classification purposes as there can be significant diversity within some Inc. groups as well as co-integration of multiple replicon types in a single plasmid (Partridge et al., 2018; Hsu et al., 2019).

**Figure 1:**
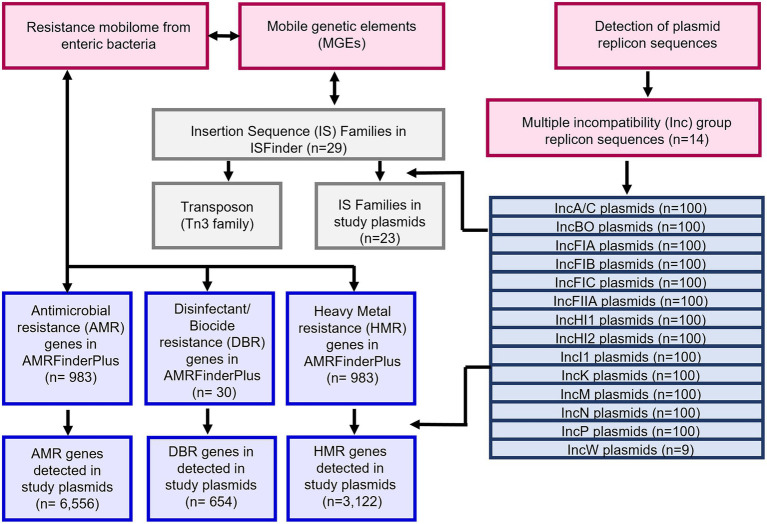
Workflow of the study to examine the resistance gene and MGE content of multiple plasmids representing those plasmid types most commonly associated with carrying antimicrobial resistance (AMR) genes in enteric bacterial pathogens.

The distribution and genetic identification of resistance genes among individual plasmid types across different bacterial strains have been documented (Venturini et al., 2013; Carattoli et al., 2015; Reid et al., 2015; Seiffert et al., 2017; Moran and Hall, 2019; Chang et al., 2020; Majewski et al., 2021). In the current study, we applied several bioinformatic tools including BLAST, AMRFinderPlus, and ISFinder for detecting genes in assembled plasmid sequence data and assessed the combination of resistance genes and MGEs, and individual plasmid types from enteric bacterial species to better elucidate AMR epidemiology as an effort to respond to this important public health threat. Understanding the AMR mobilome is important for the development of strategies that can limit the transfer of resistant pathogens to humans via contaminated foods (Johansson et al., 2021; Algarni et al., 2022). A schematic of the flow of the experiments is provided in Figure 1.

## Methods and materials

### Selection of enteric plasmids for sequence analyses and data acquisition

The GenBank database was screened for 14 different plasmid replicon type sequences: A/C, B/O, FIA, FIB, FIC, FIIA, HI1, HI2, I1, L/M, N, P and W (Carattoli, 2009; Rozwandowicz et al., 2018). To select the sequences for further analyses, representative plasmid replicon sequences described by Carattoli et al. (2005) were used for BLAST searching (Supplementary Table S1). Individual replicon sequences were queried by selecting “Microbe” genomes and “Complete plasmids” in the Microbial Nucleotide BLAST database using default settings (i.e., optimize for “highly similar sequences,” megablast). These queries were conducted in January 2022. The DNA sequences for the initial set of 100 plasmids identified for each replicon type were downloaded as a FASTA file (with the exception of IncW, where there were only 9 sequences available using the search parameters for the study). This set of extracted sequences provided 1,309 plasmids to assess the presence of resistance genes and MGEs across the plasmid replicons. The identity and accession numbers of these plasmids in this “test” set are provided in Supplementary Table S2. In addition, to evaluate the use of 100 plasmids per group, a validation set of 563 IncA/C plasmid sequences was extracted from GenBank. This set included all of the plasmid IncA/C sequences detected at the time of data extraction using the search criteria noted above. A second set of analyses to assess how representative the sources of the plasmid subsets (test set) were to the larger plasmid data present in GenBank was completed. The description and bacterial source information for all plasmids meeting the above noted search criteria were downloaded (December of 2022) and the genera of the source organisms of this “reference” set were counted and the average percent for each taxa determined to compare to the test set used in the studies.

### Identification of resistance genes and integrons using GalaxyTrakr tools

Analyses were performed by analyzing the FASTA files for the individual replicon types described above using the latest AMRFinderPlus database V3.2.1 (available at^1^) and IntegronFinder (Néron et al., 2022). The AMRFinderPlus database has sequences for 983 unique AMR genes (including 6,060 total variants), 30 DBR genes (including 55 total variants) and 82 HMR genes (including 182 total variants). The analyses were run using the AMRFinderPlus plugin within the GalaxyTrakr^2^ operating environment to identify AMR genes, disinfectant, biocide and metal heavy resistance genes located within the submitted sequences. The input file for each replicon (Inc) type contained the sequences for 100 plasmids (with the exception of IncW, *n* = 9). A report outlining the identity and numbers of the AMR genes belonging to different antimicrobial compounds classes, as well as DBR and HMR genes, was downloaded from GalaxyTrakr and parsed out based on their functions (AMR, DBR and HMR) to allow for comparison between the different plasmid types. For integron detection, the analyses were done using the IntegronFinder within the GalaxyTrakr and the resultant summary and integron annotation files were downloaded to determine the numbers of complete integrons, Cluster of *attC* sites Lacking Integrase Nearby (CALIN) elements and Integron integrase only (In0) elements, without any *attC* site nearby (Néron et al., 2022).

### Identification of IS and Tn elements

To identify the IS and Tn elements, the combined FASTA files described above for each Inc. type were used to identify the IS elements using the ISFinder program^3^ (accessed on 02/17/2022) and transposons using the TnCentral program.^4^ The FASTA files were uploaded into either the ISFinder BLAST (blastn) or TnCentral interfaces using the default parameters. The ISFinder database included sequenced for 5,735 different IS elements that were divided amongst 29 different IS families. The output provided information on the sequences producing significant alignments based on BLAST e-value, IS family, group, score (bits) and e-value. The TnCentral database (accessed on 12/06/2022) had 433 transposable elements including transposons and composite transposons (Ross et al., 2021). The data on the transposable elements was downloaded from each plasmid type and the Tns for each plasmid type parsed out to facilitate the comparison of the distribution among the different plasmid groups.

### Statistical analyses

The majority of statistics for this project are descriptive in nature. Since for all but IncW, the denominator for the numbers of plasmids is 100, the percentages are equal to the number of positive values. For IncW and the analyses of the IncA/C validation and plasmid source reference sets, the means were calculated to facilitate comparison of the proportion of genetic elements to the test set used in the study. For comparison of the distribution of AMR, DBR, and HMR and MGE traits across the different plasmid replicon types, student *t*-tests were used with statistical significance observed at *p* < 0.05.

## Results and discussion

To explore the diversity between different plasmid types and the presence/absence AMR genes within the plasmid database, we performed AMRFinderPlus analyses to extract AMR genes and extrapolate the resistance to the antibiotic compounds that are related to each gene (output data available in Supplementary Table S3). We selected 14 plasmid replicon or Inc. types that are associated with antimicrobial resistance in enteric bacteria (Carattoli, 2009; Rozwandowicz et al., 2018). From each Inc. type, with the exception of IncW, we used a convenience sample of 100 whole plasmid sequences (1,309 in total) for analyses. These plasmids originated from members of the Gammaproteobacteria, with the great majority being from members of the Enterobacteriaceae family and a small number from the *Morganellaceae* and *Vibrionaceae* families (Supplementary Table S2).

### AMR gene analysis

With AMRFinderPlus, 195 unique resistance genes were detected in at least one of the 1,309 sequences screened, with the distribution of genes varying across each of the plasmid types (Supplementary Table S4 and Table 1). Among the different plasmid types, IncHI2 (*n* = 1,033), N (*n* = 932), A/C (*n* = 820), P (*n* = 733), HI1 (*n* = 732), and FIA (*n* = 574) exhibited the highest abundance of AMR genes. Regarding the diversity of the resistance genes (number of unique genes/total number of genes detected) the IncFIA plasmids had lowest diversity (0.06; 34 unique genes/574 genes detected). Indeed, several IncFIA resistance genes were detected in at least 48 percent of the plasmids, including *aadA5, aph(3″)-Ib, aph(6)-Id, bla*_CTX-M-27_*, dfrA17, mph(A), sul1, sul2*, and *tet*(*A*; range is *n* = 48–55; Table 1). In contrast, the IncW plasmids showed the highest calculated diversity of resistance genes (0.68), which was driven by the observation that there was a cumulative total of 34 genes detected, representing 23 distinct genes. It is important to note that for IncW, only nine plasmids were screened, which likely contributed to the high perceived level of diversity as there may have been a bias in the plasmids that were sequenced due to the carriage of resistance genes. Among those with 100 sequences analyzed, the IncFIIA had the highest calculated diversity (0.34) of resistance genes, which was driven by the low numbers of total genes detected (N = 82).

**Table 1:** Numbers of representative^A^ antimicrobial resistance genes detected in 100^B^ sequences for each plasmid replicon type identified using AMRFinderPlus.

AMR Genes	Products	Resistance genes compounds	IncA/C	IncBO	IncFIA	IncFIB	IncFIIA	IncFIC	IncHI1	IncHI2	IncI1	IncK	IncM	IncN	IncP	IncW^B^
*aac(3)-IId*	aminoglycoside *N*-acetyltransferase AAC(3)-IId	Gentamicin	12		17	6		6	23	7	4		1	6	7	
*aac(3)-IIg*	aminoglycoside *N*-acetyltransferase AAC(3)-IIg	Gentamicin								20				2		
*aac(6’)-Ib3*	aminoglycoside *N*-acetyltransferase AAC(6’)-Ib3	Amikacin/Kanamycin/ Tobramycin	36			3				13			8	1		
*aac(6’)-II*	aminoglycoside *N*-acetyltransferase AAC(6’)-IIc	Aminoglycoside	7							28			1	2		
*aadA1*	ANT(3’’)-Ia family aminoglycoside nucleotidyltransferase AadA1	Gentamicin	23	9	2	3	2	5	20	27	20	9	16	26	60	
*aadA2*	ANT(3’’)-Ia family aminoglycoside nucleotidyltransferase AadA2	Streptomycin	29	2	1	13	2	6	16	34	13	1	3	29	26	1
*aadA5*	ANT(3’’)-Ia family aminoglycoside nucleotidyltransferase AadA5	Streptomycin			48	13		4	10	2			1	9	3	
*aph(3’)-Ia*	aminoglycoside O-phosphotransferase APH(3’)-Ia	Sulfonamide	12	8	3	2	2	10	23	17	3	3	7	24	29	
*aph(3’’)-Ib*	aminoglycoside O-phosphotransferase APH(3’’)-Ib	Streptomycin	14	22	53	20	8	16	49	47	7	17	40	41	45	2
*aph(6)-Id*	aminoglycoside O-phosphotransferase APH(6)-Id	Streptomycin	20	22	54	20	8	1	49	47	7	17	11	41	43	2
*armA*	ArmA family 16S rRNA (guanine(1405)-N(7))-methyltransferase	Gentamicin	20	1					1	1		1		4	1	
*arr*	NAD(+)--rifampin ADP-ribosyltransferase	Rifamycin								20			1	3		
*bla*CMY-2	class C β-lactamase CMY-2	Cephalosporin	6	13					1		20	14			5	
*bla*CMY-6	class C β-lactamase CMY-6	Cephalosporin	34			1										
*bla*CTX-M-14	class A extended-spectrum β-lactamase CTX-M-14	Cephalosporin	2	2	2	3	1	3	1	2	10	6	20	7		
*bla*CTX-M-27	class A extended-spectrum β-lactamase CTX-M-27	Cephalosporin			48										1	
*bla*NDM-1	subclass B1 metallo-β-lactamase NDM-1	Carbapenem	54			3			1	4				1		1
*bla*OXA-48	carbapenem-hydrolyzing class D β-lactamase OXA-48	β-Lactam											20			
*bla*SHV-12	class A extended-spectrum β-lactamase SHV-12	β-Lactam	1							30			4	1		
*bla*TEM-1	class A broad-spectrum β-lactamase TEM-1	β-Lactam	20	26	31	20	15	16	60	37	8	20	23	44	56	1
*ble*	bleomycin binding protein Ble-MBL	Bleomycin	52		1	9		4	7	3	3			14	3	2
*bleO*	bleomycin binding protein	Bleomycin				1		5	4	1			1	23	3	
*catA1*	type A-1 chloramphenicol O-acetyltransferase	Chloramphenicol	2	3	2			2	41	15		2	3		14	
*catA2*	type A-2 chloramphenicol O-acetyltransferase CatII	Chloramphenicol	4				4			30			1	4		
*cmlA1*	chloramphenicol efflux MFS transporter CmlA1	Chloramphenicol				3	1		8	2	10		1	20	11	
*dfrA1*	trimethoprim-resistant dihydrofolate reductase DfrA1	Trimethoprim	8	4		2		2	2	4	2	4	1	1	27	
*dfrA17*	trimethoprim-resistant dihydrofolate reductase DfrA17	Trimethoprim			48	16		7	10	1				9	3	
*dfrA19*	trimethoprim-resistant dihydrofolate reductase DfrA19	Trimethoprim								26			1			
*dfrA7*	trimethoprim-resistant dihydrofolate reductase DfrA7	Trimethoprim					3		20						1	
*ere(A)*	EreA family erythromycin esterase	Trimethoprim								21				2		
*floR*	chloramphenicol/florfenicol efflux MFS transporter FloR	Chloramphenicol	3	1		7	1	9	31	4	6	1	2	36	12	1
*fosA3*	fosfomycin resistance glutathione transferase FosA3	Fosfomycin				2		4	2	3	1			23	5	
*mcr-9.1*	phosphoethanolamine--lipid A transferase MCR-9.1	Colistin								64			1			
*mph(A)*	Mph(A) family macrolide 2’-phosphotransferase	Macrolide	16	3	55	21		7	12	10	3	2	3	34	16	
*oqxB*	multidrug efflux RND transporter permease subunit OqxB	Quinolone				1		4	1	1				20	2	
*qnrA1*	quinolone resistance pentapeptide repeat protein QnrA1	Sulfonamide	14							23			1			
*qnrS1*	quinolone resistance pentapeptide repeat protein QnrS1	Quinolone	1	3		2	1	4	26	5	1	3	5	16	2	
*rmtC*	RmtC family 16S rRNA (guanine(1405)-N(7))-methyltransferase	Aminoglycoside	34			1				1						
*sul1*	sulfonamide-resistant dihydropteroate synthase Sul1	Sulfonamide	121	9	54	26	4	8	40	147	9	8	34	34	74	6
*sul2*	sulfonamide-resistant dihydropteroate synthase Sul2	Sulfonamide	7	29	53	19	6	17	49	18	5	21	4	44	32	
*sul3*	sulfonamide-resistant dihydropteroate synthase Sul3	Sulfonamide				4	1	5	14		10			22	12	
*tet(A)*	tetracycline efflux MFS transporter Tet(A)	Tetracycline	5	13	54	44	8	20	13	18	9	8	7	55	100	1
*tet(B)*	tetracycline efflux MFS transporter Tet(B)	Tetracycline	1	9	2	5		4	58	18	3	7	2		1	
*tet(D)*	tetracycline efflux MFS transporter Tet(D)	Tetracycline	2							25				3		

A This table includes those resistance genes that for found in at least 20% of one or more of the plasmid types. The complete table all resistance genes is Supplementary Table S4.

B For IncW there were only nine plasmids that met the inclusion criteria and were analyzed.

^C^The colors are based on a progression of the numbers of specific genes detected in a plasmid type moving from a darker green for the lowest number (1) detected to lighter green to yellow to orange and red for the highest number of genes.

^D^Please note that some plasmids have more than one copy of an individual gene, which can lead to having numbers greater than 100.

An interesting observation among some of the genes was that there were greater than 100 *sul1* genes in the IncA/C (*n* = 121) and the IncHI2 (*n* = 127) plasmids and 100 *tet(A)* genes detected in the IncP plasmids (Table 1). The sizes of these plasmid types are generally large, with some of the IncHI2 and IncP plasmids over 400 KB in size (e.g., GenBank accession numbers NZ_CP043927.1 and NZ_MN256757.1, respectively) and IncA/C plasmids routinely over 100 KB in size with multiple resistance operons (Harmer and Hall, 2014). When the numbers of unique plasmids carrying the resistance genes were evaluated in these cases, there were 80 IncA/C and 78 IncHI2 plasmids with a *sul1* gene and 99 IncP plasmids with *tet*(*A*; Supplementary Table S4).

### DBR and HMR gene analyses

Further, we used the AMRFinderPlus tool to characterize the DBR and HMR genes across the plasmid replicons to count the number of each gene present in the dataset. We identified 5 unique biocide resistance genes, with the highest cumulative numbers being for IncHI2 (*n* = 146), A/C (*n* = 115), P (*n* = 90), FIA (*n* = 58), M (*n* = 58) and HI1 (*n* = 54; Table 2). By far the most common biocide resistance gene was *qacE*Δ1, which accounted for 464/654 (70.9%) of the DBR genes. For the HMR, 40 unique genes were detected among the strains tested (Table 3). One of the key features of the HMR genes is that they often occur as part of multigene operons, including arsenite efflux (*ars* genes), mercury resistance (*mer* genes), nickel resistance (*ncr*), copper resistance (*pco* genes), copper/silver resistance (*sil* genes), and tellurium resistance (*ter* genes; Table 3). The highest numbers of HMR genes were found in the IncHI2 (*n* = 1,166), P (*n* = 639) and HI1 (*n* = 404) plasmids. As was observed with the AMR genes, the IncP and HI2 and I1 plasmids have greater than or very near counts of 100 for some of the HMR genes. For example, with the IncP plasmids, three of the genes in the mercury resistance operon (*merP, R* and *T*) have between 103 and 110 gene copies, while for IncHI2 plasmids there were 97 *merR* and *terD* and 99 *terW* and *terZ* genes detected (Table 3).

**Table 2:** Disinfectant and biocide resistance genes and integron-associated elements detected in 100^A^ representative sequences for each plasmid replicon type identified using AMRFinderPlus or IntegronFinder, respectively.

Gene/ element	Products	IncA/C	IncBO	IncFIA	IncFIB	IncFIIA	IncFIC	IncHI1	IncHI2	IncI1	IncK	IncM	IncN	IncP	IncW^A^
*qacE*	Quaternary ammonium compound efflux SMR transporter QacE	29^B^		4	2			1	47	1		4	13	5	2
*qacE*Δ*1*	Quaternary ammonium compound efflux SMR transporter QacE delta 1	82	9	54	26	4	10	39	96	9	8	30	23	68	6
*qacG2*	Quaternary ammonium compound efflux SMR transporter QacG2								1				1	5	1
*qacL*	Quaternary ammonium compound efflux SMR transporter QacL	4			3	1	4	14		11		2	21	12	
*smr*	Multidrug efflux SMR transporter Smr														
Complete Integron	Integron with integron integrase nearby attC site(s)	96	6	45	29	14	7	66	105^C^	27	11	39	51	87	6
CALIN	Cluster of attC sites lacking integrase nearby	2	1	5	5	4		8	9	1	4	2	19	9	
In0	Integron integrase only, without any attC site nearby	10		6	9	13	1	8	46			1	20	6	

A For IncW there were only nine plasmids that met the inclusion criteria and were analyzed.

B The colors are based on a progression of the numbers of specific genes detected in a plasmid type moving from a darker green for the lowest number (1) detected to lighter green to yellow to orange and red for the highest number of genes.

C Please note that some plasmids have more than one copy of an individual gene.

**Table 3:** Heavy metal resistance genes detected in 100^A^ representative sequences for each plasmid replicon type identified using AMRFinderPlus.

Metal resistance genes	Products	IncA/C	IncBO	IncFIA	IncFIB	IncFIIA	IncFIC	IncHI1	IncHI2	IncI1	IncK	IncM	IncN	IncP	IncW^A^
*arsA*	arsenite efflux transporter ATPase subunit ArsA	6^B^						1				2	1		
*arsB*	arsenite efflux transporter membrane subunit ArsB	5						1				2	1		
*arsC*	glutaredoxin-dependent arsenate reductase	5						1	81	1		3	1		
*arsD*	arsenite efflux transporter metallochaperone ArsD	7						9				2	1		
*arsR*	arsenite efflux transporter ATPase subunit ArsR	7						9				2	1		
*klaB*	tellurium resistance system protein klaB													2	
*klaC*	tellurium resistance system protein klaC													2	
*merA*	mercury(II) reductase	13						1	60			25		14	1
*merB*	organomercurial lyase MerB	11							20			2	1	14	
*merC*	organomercurial transporter MerC	14	16	3	4	1	4	50	17	9	11	1	11	89	
*merD*	mercury resistance co-regulator MerD	13						7	75	1		23	1	15	1
*merE*	broad-spectrum mercury transporter MerE	13						7	59			21		12	1
*merG*	phenylmercury resistance protein MerG								3						
*merP*	mercury resistance system periplasmic binding protein MerP	25	16	3	4	1	4	56	24	8	11	2	8	103^C^	1
*merR*	mercury resistance transcriptional regulator MerR	57	16	2	6	1	5	60	97	9	11	29	13	110	1
*merT*	mercuric transport protein MerT	29	16	3	4	1	4	60	80	9	11	27	12	106	1
*ncrA*	Metal Resistance								1						
*ncrB*	nickel-sensing transcriptional repressor NcrB								1						
*ncrC*	Ni(II)/Co(II) efflux transporter permease subunit NcrC								1						
*pcoA*	multicopper oxidase PcoA	1		1				7	9	1			9	8	
*pcoB*	copper-binding protein PcoB	1		1				7	8	1			4	8	
*pcoC*	copper resistance system metallochaperone PcoC	1		1				7	9	1			5	8	
*pcoD*	copper resistance inner membrane protein PcoD	1		1				2	8	1			1	8	
*pcoE*	copper resistance system metallochaperone PcoE	1						5	8				5	6	
*pcoR*	copper response regulator transcription factor PcoR	1		1				2	8	1			1	8	
*pcoS*	copper resistance membrane spanning protein PcoS	1		1				8	82	1		1	5	8	
*silA*	Cu(+)/Ag(+) efflux RND transporter permease subunit SilA	1		1	2			9	26	1			12	8	
*silB*	Cu(+)/Ag(+) efflux RND transporter periplasmic adaptor subunit SilB	1		1	2			9	26	1			12	8	
*silC*	Cu(+)/Ag(+) efflux RND transporter outer membrane channel SilC	1		1	2			9	26	1			12	8	
*silE*	silver-binding protein SilE	1		1	2			9	29	1			12	8	
*silF*	Cu(+)/Ag(+) efflux RND transporter periplasmic metallochaperone SilF	1		1	2			9	26	1			12	8	
*silP*	Ag(+)-translocating P-type ATPase SilP	1		1	2			9	29	1			11	8	
*silR*	copper/silver response regulator transcription factor SilR	1		1	2			9	30	1			12	8	
*silS*	copper/silver sensor histidine kinase SilS	1		1	2			9	29	1			12	8	
*terB*	tellurium resistance membrane protein TerB	1		1				8		1			2		
*terC*	tellurium resistance membrane protein TerC	1		1				8		1			2		
*terD*	tellurium resistance membrane protein TerD	1		1		2	1	8	97	2		1	34	18	
*terE*	tellurium resistance membrane protein TerE	1		1				8		1			2		
*terW*	tellurium resistance protein TerW					2	1		99			1	32	18	
*terZ*	tellurium resistance-associated protein TerZ					2	1		99	1		1	32	18	

A For IncW there were only nine plasmids that met the inclusion criteria and were analyzed.

B The colors are based on a progression of the numbers of specific genes detected in a plasmid type moving from a darker green for the lowest number (1) detected to lighter green to yellow to orange and red for the highest number of genes.

C Please note that some plasmids have more than one copy of an individual gene.

### Comparison of resistance genes

**Figure 2:**
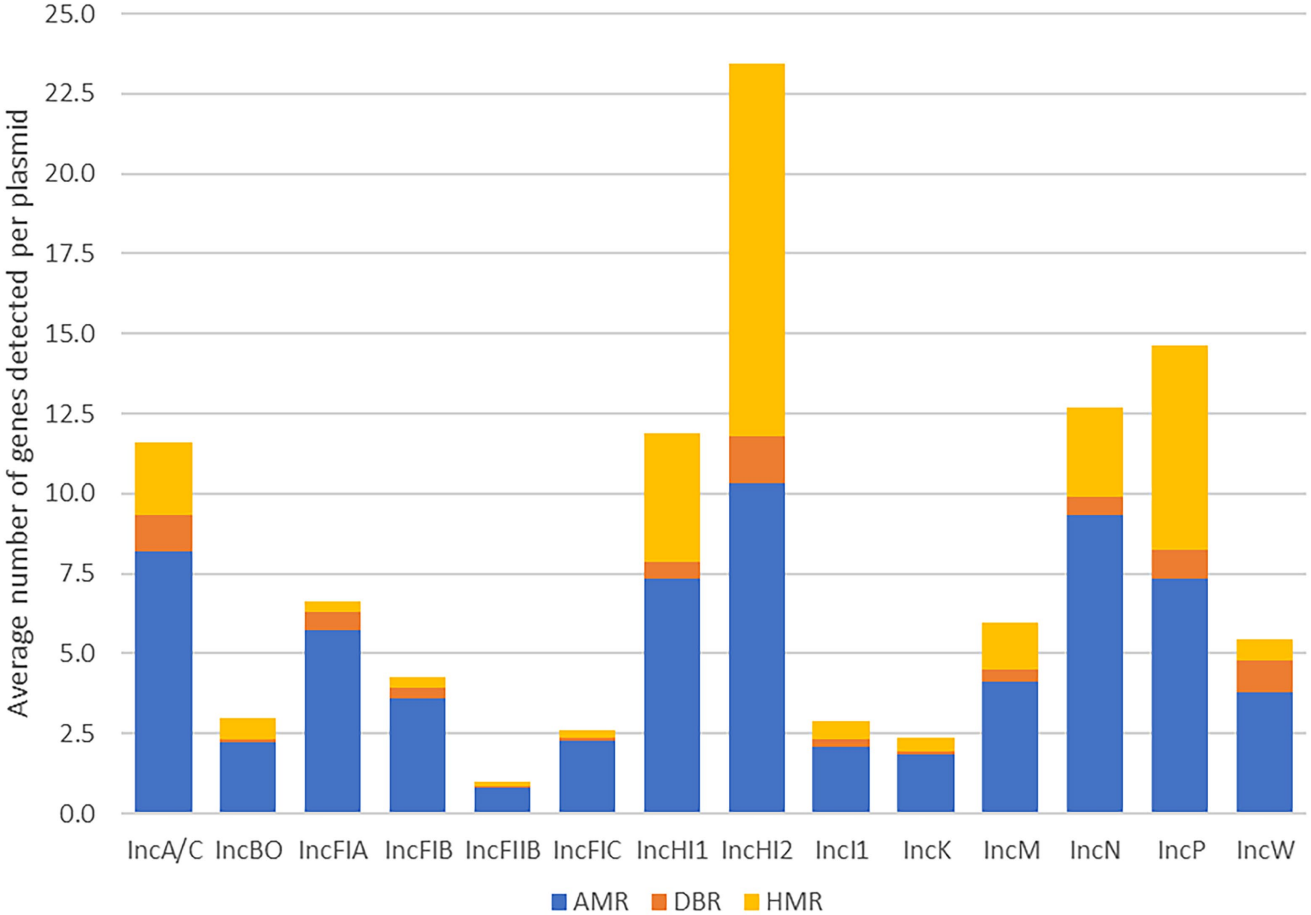
Average number of antimicrobial resistance (AMR), disinfectant and biocide resistance (DBR) and heavy metal resistance (HMR) genes detected per plasmid in each of the plasmid replicon types.

We next compared the pattern of the data of three types of resistance genes including AMR, DBR and HMR to determine the more global diversity of resistance genes across the 14 plasmid types associated with enteric bacteria (Table 4; Figure 2). When the percentage the three resistance gene types across of each plasmid replicons was evaluated in relation to the total numbers of acquired resistance genes in the AMRFinderPlus database, the results were variable across the different plasmid types, with the overall average percentage of AMR, DBR, and HMR genes detected being 5.5, 9.0, and 22.5%, respectively (Table 4).

**Table 4:** Relative percentage of the different gene types compared to the number of corresponding genes in AMRFinderPlus (AMR: *n* = 983; Biocide: N = 30; Metal: N = 82) and ISFinder (N = 29).

Replicon types	Percent of genes present among plasmid replicons				Average resistance genes	*p* value
	AMR	Biocide	Metal	IS/Tn families		
A/C	7.2	10.0	39.0	58.6	18.7	0.30
BO	4.2	3.3	4.9	62.1	4.1	0.09
FIA	3.5	6.7	26.8	65.5	12.3	0.50
FIB	4.6	10.0	14.6	62.1	9.7	0.34
FIIB	2.8	6.7	8.5	58.6	6.0	0.16
FIC	4.7	6.7	8.5	65.5	6.6	0.17
HI1	6.7	10.0	37.8	65.5	18.2	0.31
HI2	8.9	13.3	37.8	58.6	20.0	0.25
I1	4.5	10.0	30.5	62.1	15.0	0.40
K	4.2	3.3	4.9	62.1	4.1	0.09
M	7.7	10.0	20.7	58.6	12.8	0.47
N	9.2	13.3	39.0	62.1	20.5	0.24
P	6.1	13.3	34.1	55.2	17.9	0.30
W	2.3	10.0	7.3	55.2	6.6	0.18
Average	5.5	9.0	22.5	60.8	12.3	

Average resistance gene percentages highlighted in red font had > 15% of genes detected and discussed further in the body of the manuscript.

Likewise, according to the overall dataset it showed that there were six plasmid types with relatively high prevalence of resistance genes (≥15% percent average) compared to other individual plasmid types, which these included IncN (20.5%), HI2 (20.0%), A/C (18.7%), HI1 (18.2%), P (17.9%), and I1 (15.0%; Table 4, highlighted in red text). These observed elevated average resistance gene levels were largely due to the HMR genes, as those with the highest overall percentages had the highest metal gene percentages; however, many of these types also had higher than average AMR gene percentages as well. In contrast, the IncBO and K plasmids had the lowest numbers of unique resistance genes detected compared to the other plasmid types, however none of the differences in resistance gene content between plasmid types reached statistical significance.

The association of the common resistance genes across the different Inc. groups may suggest mobility of AMR, DBR, and HMR within bacterial populations through plasmid transfer (Tamminen et al., 2012). These observations further indicate the importance of plasmid transfer for the dissemination of resistance across the Enterobacteriaceae (Rodrigues et al., 2020; Che et al., 2021; Li et al., 2021). Moreover, previous studies also showed the prevalence of AMR, DBR and HMR in *S. enterica* isolates was mainly plasmid mediated (Mourao et al., 2015; Deng et al., 2018). Looking specifically at the individual plasmid types with the highest prevalence of resistance genes (IncN, HI2, I1, HI1, P and A/C), there are some key features. For example, the IncA/C plasmids have been isolated from multiple *S. enterica* and *E. coli* strains associated with infections of animals and human patients and often found to simultaneously contain multiple AMR, DBR and HMR genes (Harmer and Hall, 2014; Mattioni Marchetti et al., 2020; Octavia et al., 2020; Zhao et al., 2020). The IncI1-complex of plasmids also has a global diversity in Enterobacteriaceae, and many representatives carry multiple resistance genes, including those for clinically important agents, and they have been reported as a major driver of AMR in *Salmonella, E. coli* and *Klebsiella pneumoniae* (Sekizuka et al., 2017; Abraham et al., 2018; Zhang et al., 2019; Foley et al., 2021; Oladeinde et al., 2021). The IncHI plasmids tend to be very large plasmids, often greater than 200 kB in size and carry multiple AMR, HMR and DBR operons, as well as virulence factors that are important for enteric fever (Phan and Wain, 2008; Han et al., 2012). The IncN and IncP plasmids have relatively broad host ranges and are found to disseminate AMR, which has been demonstrated in *K. pneumoniae, E. coli* and *S. enterica* isolated from healthy humans and clinically ill patients (Wailan et al., 2015; Lu et al., 2017). These diverse groups of medically important plasmids have shown the utility of sequence data in characterizing plasmids of Gram-negative bacteria and the use of sequencing has been proposed as a method to assess the functions of plasmids to improve health outcomes (Harmer and Hall, 2014).

### IS element, Tn and integron analyses

To assess the presence of IS elements, Tns and integrons in the sequenced plasmids, ISFinder, TnCentral and IntegronFinder were utilized, respectively. The ISFinder program has been used for the analyses the mobilome of *S. enterica*, *E. coli*, *K. pneumoniae*, *Acinetobacter baumanii*, *Pseudomonas aeruginosa*, *Enterobacter* spp. and *Shigella* spp. which are included in the World Health Organization (WHO)‘s list of the important pathogens (World Health Organization, 2020; Zhang et al., 2022). In this study, ISFinder predicted over 250 MGEs; among these, there were 24 (from a total of 29) different families of IS/Tn3 elements identified in at least one plasmid (Table 5). The prevalence of ISs was highly diverse and variable within the data set, with 11 of 29 IS families identified in all 14 plasmid types. Among these, Tn3 was among the most abundant of the sub-families identified across all the individual plasmid replicons (Table 5). When the Tn elements were examined in broader detail using the TnCentral analyses, there were 186 different Tns identified in at least one of the plasmid types (Supplementary Table S5). The majority of the elements (*n* = 120, 65%) were detected in at least one representative plasmid in all 14 plasmid types. Additionally, integrons were widely distributed across the different plasmid types (Table 2), with several plasmids have multiple complete integrons. This phenomenon is highlighted by the fact that there were 105 complete integrons detected among the 100 IncHI2 plasmids examined (Table 2). The genetic variability and widespread distribution of the MGEs contributes to the dynamic diversity observed in the corresponding host genetics (Mahillon and Chandler, 1998; Vandecraen et al., 2017). Further study is required to evaluate the combination of resistance genes type and IS/Tn in the individual plasmid replicon types.

**Table 5:** IS families detected (brown box) in the representative plasmid types using ISFinder.

IS family	Product	IncA/C	IncBO	IncFIA	IncFIB	IncFIIA	IncFIC	IncHI1	IncHI2	IncI1	IncK	IncM	IncN	IncP	IncW
IS110	IS110 family transposase ISMav6	**X**	**X**	**X**	**X**	**X**	**X**	**X**	**X**	**X**	**X**	**X**	**X**	**X**	**X**
IS21	IS21 family transposase	**X**	**X**	**X**	**X**	**X**	**X**	**X**	**X**	**X**	**X**	**X**	**X**	**X**	**X**
IS256	Mutator IS256 family transposase	**X**	**X**	**X**	**X**	**X**	**X**	**X**	**X**	**X**	**X**	**X**	**X**	**X**	**X**
IS3	IS3 element transposase of IS3 family	**X**	**X**	**X**	**X**	**X**	**X**	**X**	**X**	**X**	**X**	**X**	**X**	**X**	**X**
IS4	IS4 family transposases	**X**	**X**	**X**	**X**	**X**	**X**	**X**	**X**	**X**	**X**	**X**	**X**	**X**	**X**
IS5	IS5 family transposase	**X**	**X**	**X**	**X**	**X**	**X**	**X**	**X**	**X**	**X**	**X**	**X**	**X**	**X**
IS6	IS6 family transposase	**X**	**X**	**x**	**X**	**X**	**X**	**X**	**X**	**X**	**X**	**X**	**X**	**X**	**X**
IS66	IS66 family transposase	**X**	**X**	**X**	**X**	**X**	**X**	**X**	**X**	**X**	**X**	**X**	**X**	**X**	**X**
ISL3	ISL3 family transposase	**X**	**X**	**X**	**X**	**X**	**X**	**X**	**X**	**X**	**X**	**X**	**X**	**X**	**X**
ISNCY	ISNCY family transposase	**X**	**X**	**X**	**X**	**X**	**X**	**X**	**X**	**X**	**X**	**X**	**X**	**X**	**X**
Tn3	Type II transposons of Tn3 family	**X**	**X**	**X**	**X**	**X**	**X**	**X**	**X**	**X**	**X**	**X**	**X**	**X**	**X**
IS630	IS630 family transposase	**X**	**X**	**X**	**X**	**X**	**X**	**X**	**X**	**X**	**X**	**X**	**X**	**X**	
IS30	IS30 family transposase IS1062	**X**	**X**	**X**	**X**		**X**	**X**	**X**	**X**	**X**	**X**	**X**	**X**	**X**
ISKra4	ISKra4 family transposases	**X**	**X**	**X**	**X**	**X**	**X**	**X**	**X**	**X**	**X**	**X**	**X**		**X**
IS481	IS481 family transposase		**X**	**X**	**X**	**X**	**X**	**X**	**X**	**X**	**X**	**X**	**X**	**X**	
IS1182	IS5/IS1182 family transposase	**X**		**X**	**X**	**X**	**X**	**X**	**X**	**X**		**X**	**X**	**X**	**X**
IS1	IS1 family transposase	**X**	**X**	**X**	**X**	**X**	**X**	**X**			**X**	**X**	**X**		**X**
IS200/IS605	IS200/IS605 family transposase		**X**	**X**	**X**	**X**	**X**	**X**		**X**	**X**		**X**	**X**	**X**
ISAs1	ISAs1 family transposase	**X**	**X**	**X**			**X**	**X**	**X**	**X**	**X**				
IS91	IS91 family transposase		**X**	**X**		**X**	**X**		**X**	**X**					**X**
IS1380	IS1380 family transposase ISEc9			**X**		**X**			**X**				**X**		**X**
IS1634	IS1634 family transposase IS1549							**X**				**X**		**X**	
ISAzo13	ISAzo13-like element ISCfu1 family transposase														**X**
ISH6	ISH6 family transposase														**X**
IS1595	IS1595 family transposase														
IS701	IS701 family transposase														
ISH3	ISH3 family transposase														
IS607	IS607 family transposase														
IS982	IS982 family transposase														

To gain a fuller understanding of the distribution of the MGEs across the plasmid types, a comparison was performed between resistance gene elements and MGEs (IS/Tn) in the plasmid data set was evaluated. As noted above, six replicons including IncA/C, I1, HI1, HI2, N, and P possessed the highest number of different resistance genes and MGEs as determined by calculating the percentage of each in individual plasmid replicons (each had an average of at least 15% presence among the different resistance groups; Table 4). When the numbers of different families of IS and Tn elements were detected, these plasmid types did not have a significantly increased number of IS families (Table 5). The overall range of IS families was 17 to 21 per plasmid type (average 18.9; SD 1.1); among the six replicons, the range was 17 to 20 (average 18.7; SD 1.1), thus the increase in numbers of genes was not due to an increase in IS/Tn element. With the Tn analyses, the overall numbers detected among the different plasmid types ranged from 135 (IncFII) to 172 (IncN; average 152.7; SD 10.8; Supplementary Table S5). Among the six replicons noted above, the numbers of Tns detected ranged from 151 to 172, with an average of 160.7 (SD 8.9), thus this group did not have a significantly higher number of Tns than the plasmid population as a whole.

Several of the HMR elements are part of the larger set of operons (*mer, ars, pco, sil*, and *ter*) with multiple genes per operon, which led in part to IncA/C, I1, HI1, HI2, N, and P plasmids having the highest percentage of genes (Tables 3, 4). These HMR operons are typically part of a IS element or Tn on a plasmid, thus the increase in genes does not lead to a proportional increase in IS/Tn elements (e.g., unlike AMR, where a single gene leads to resistance, for HMR multiple genes in an operon are required to encode the resistance; Han et al., 2012).

Because these MGEs are often located on plasmids, it is reasonable to believe that plasmids are required components for understanding the dissemination of resistance genes in isolates from food producing animals and humans (Wang et al., 2014). It has been reported the MDR regions carried on different plasmids can be present in novel MGEs in clinical bacterial strains (Cheng et al., 2019). For example, MGEs located on transmissible plasmids, particularly IncI and IncF groups, have been previously demonstrated to facilitate resistance transmission (Venturini et al., 2013). The Tn3-type Tns plays a critical role in the evolution of both MDR plasmids and chromosomal islands in the Enterobacteriaceae (Venturini et al., 2013). Several conserved and variable resistance gene type (RGs)-MGEs combinations have been observed across unrelated enteric pathogens and were located on different individual plasmid replicons based on the results of the current study. In our data, there was a significant overlap in the presence of the genes *sul1, qacE*Δ1 and complete integrons, which was not too surprising since these resistance genes are common parts of 3′-conserved region of class 1 integrons (Supplementary Table, Table 1; Gillings et al., 2008). These integrons are also associated with a variety of IS elements that allow for the accumulation of AMR gene cassettes with in the integrons (Gillings et al., 2008, 2009). Moreover, a composite transposon-like structure carrying multiple resistance genes has been identified on various plasmid Inc. groups and in genomic islands identified in *Salmonella* and *E. coli* isolates from diverse food, animal human and environment sources (Reid et al., 2015).

**Figure 3:**
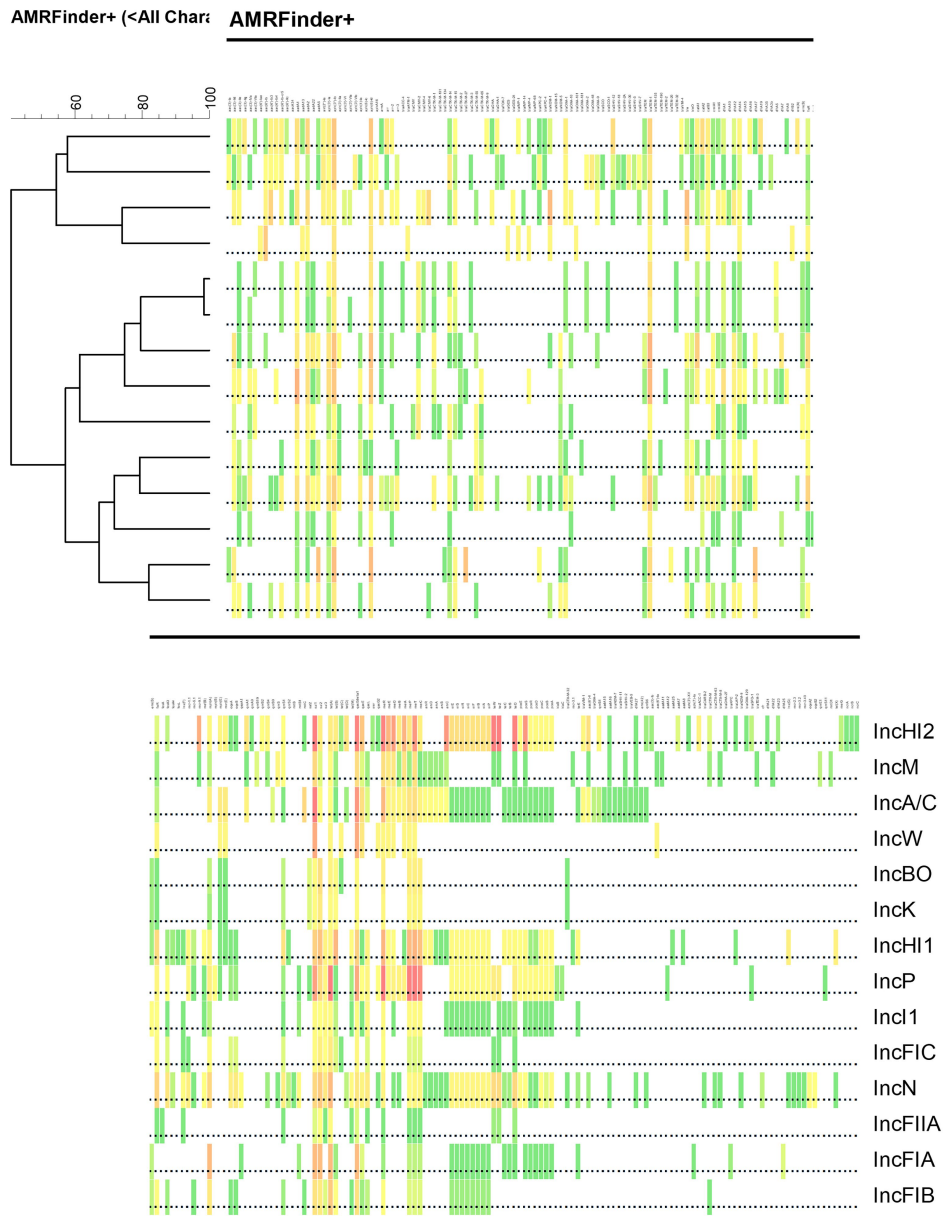
Illustration of clustering heat map association between different replicon type based on a combination of antimicrobial resistance (AMR), disinfectant and biocide resistance (DBR) and heavy metal resistance (HMR) data from Tables 1–3, respectively. Clustering analysis was performed using Pearson correlation of numerical values using BioNumerics software to generate the dendrogram using the unweighted pair group means with averages (UPGMA) algorithm. The colors of the boxes correspond to those in the tables.

One of the distinctive findings of this study, which is highlighted in Table 5, is that, among the different families of IS elements, these elements are widely distributed across the different plasmid types. Indeed, 18 of the 24 (75%) different IS families were found in at least 75% (*n* > 10/14) of the plasmid types, with 11 (45.8%) IS families being detected in all of the different plasmid types. Similar findings were also observed among the Tn elements, with the majority (65%) being detected in all 14 plasmid types. This widespread dissemination of these MGEs likely provides opportunities for the wide distribution of resistance genes associated with the elements to spread between the different plasmid types when they are co-resident within a host bacterium. The distribution of the resistance genes is further evident in the heat map that was produced to illustrate the similarities and differences of AMR, DBR and HMR genes detected across the different plasmid replicons (Figure 3). The heat map was used to cluster the groups based on the resistance gene types. According to the analysis, IncB/O and IncK are most closely related as are IncFIA and IncFIB plasmids based on the cumulative composition of resistance genes (Figure 3). These particular plasmid types have previously been shown to have similar transfer functions as well (Shirakawa et al., 2020; Zhao et al., 2020).

The use of databases and *in silico* tools allow for the prediction of bacterial function. Resistance gene prediction databases for plasmid sequences have given insights into to human health care challenges in a timely fashion (Darphorn et al., 2021). Previously, it was complex and time-consuming to characterize large populations of *S. enterica* and other enteric pathogens by their plasmid replicon typing in a laboratory setting. However, in the present *in silico* study, identifying resistance gene types and MGEs at the same time became possible and facilitated the characterization of over that 1,300 plasmid sequences from enteric pathogens that contained representative plasmid replicon types and different gene types. Indeed, the epidemiological observation of the presence of diverse MGEs suggests extremely broad dynamic potential for transfer across the range of plasmids (Che et al., 2021). Our data analysis results provide an extremely helpful way to analyze the plasmid replicon sequences as part of molecular epidemiology techniques.

A major limitation of our research is the need for extensive systemic validation of the data processing. For example, we used 100 representative plasmid sequences in the test set for each plasmid type; the question may be raised of how representative these are to the entire plasmid population. One way that this was assessed was through the comparison of the source genera of test set to the reference set that contained all of the corresponding complete plasmids in GenBank (as of December 2022). The number of available plasmids (outside of IncW) ranged from 164 (IncHI1) to 1969 (IncFIC; Supplementary Figure 1). In all cases, except for IncN, the most common source in the test set was also the most common genus in the Reference set (Supplementary Figure 1). In the case of IncN, *Klebsiella* was the common taxa in the Reference set, while *Escherichia* was the most common in the test set and second most common in the reference set. To representativeness of the gene-level data, we conducted an analysis to determine the potential impact of selection of the 100 plasmids vs. the total available at the time of selection. To do this analysis, we compared the 100 IncA/C plasmid sequences that were returned through GenBank with the total number that were available at the time (validation set, n = 563). With the validation set, 214 AMR genes were identified compared to 71 for the test set (Supplementary Table S4; Validation tab). Of the 143 genes that were not detected in the test set, but present in the validation set, 51 (36%) were present in only one plasmid and 104 (73%) were present in 5 or less plasmids, which would correspond to less than 1% of the 563 total plasmids in the validation set. These lower abundance genes are at a threshold below the 1%, which is the equivalent of a single positive in a set of 100. There were 12 (8%) instances where a gene was detected in at least 2% of the validation set, but not detected in the test set, with a range of 2.1–6.8%. This largest percentage was for *aac(6′)-Ib4*, for which some of the related variants of *aac(6′)-Ib* were more common in the test set. There were some other variabilities in the genes that were detected among the test and validation sets, with the gene *bla*_NDM-1_ detected in 54% of the test set isolates compared to 20% of the validation set. There were six additional genes that were detected in at least 20% of the test, that were present at least at a 10% lower level in the validation set, including *ble, rmtC, blaCMY-6, aac(6′)-Ib3, sul1*, and *armA*. Conversely, *sul2* was detected in 7% of the test set plasmids and 66% of the validation set isolates. There were also six additional genes that were detected in at least 20% of the validation set, but at least at a 10% lower level in the test set, including *bla*_TEM-1_, *bla*_CMY-2_, *aph(6)-Id, tet(A), floR*, and *aph(3″)-Ib*. Altogether, of the 214 AMR genes, 189 (88%) had less than 5% difference in detection rates between test and validation sets and 158 (74%) were within 2% variance of one another. Thus overall, the findings from the test set of 100 plasmid sequences was in general representative of the larger validation set of plasmids; even in cases where specific genes were much more commonly detected in the validation set, they were still detected in the test set, albeit at a lower percentage.

Another caveat with the study is that there may be a bias to resistance-related plasmids, as these have a potential be more important to public health due to their clinical relevance and may be targeted for WGS compared to those not associated with AMR. This limitation will likely diminish with the increasing availability of high-quality closed plasmids that are becoming available as the cost and technology improves. It is interesting to note that when the IncA/C validation set was downloaded in January 2022 there were 563 complete plasmids compared to 662 when the reference set was downloaded in December 2022, which represents a 17.5% increase in the available sequences in less than a year. Future prospects of our research include the expansion of the current results to look at more expansive sets of data for each of the plasmid types. These data could include assessing virulence genes carried on the plasmids. One of the features of AMRFinderPlus analyses is the detection of multiple *E. coli*-associated virulence genes and it was interesting to note that several of the plasmids in the study carried putative virulence genes (Supplementary Table S3), which can be problematic when the plasmids can encode for both increased virulence and AMR. Additionally, it may be important to have a greater detailed assessment of all accessory genes on plasmids including *mob* and *tra* genes that impact the dissemination of MGEs among enteric pathogens. These detailed analyses could also include a detailed linkage analyses of resistance genes to specific MGEs that will give more detail to the distribution of specific genetic elements through bacterial populations. This examination of the distribution of resistance genes could also look at the ICEs which can play a key role in resistance gene transmission (Johnson and Grossman, 2015; Algarni et al., 2022). As with the plasmids, the primary focus of the paper, ICEs can carry genes encoding AMR, DBR and HMR and a type IV secretion system that facilitate conjugal transfer of resistance genes (Arai et al., 2019). While they share many similarities with plasmids, ICEs are typically integrated in the host chromosome and encode for their own excision and integration into the host chromosome needed for efficient conjugal transfer. Within the ICEs, there can be other MGEs, including IS elements and Tns, that can potentially enable resistance gene transfer between ICEs and plasmids if they are co-resident in a bacterial host (Johnson and Grossman, 2015).

## Conclusion

In conclusion, the study demonstrated that AMR, DBR and HMR genes were widely distributed among the Enterobacteriaceae regardless of the source origin. Based on the *in silico* analyses, RG-MGE combinations likely demonstrate the dynamic interactions that contribute to the plasticity of multidrug-resistant pathogens and to the mobility of genes among plasmids. Overall, these findings advance the level of current understanding of the interplay of resistance genes, IS elements, plasmid replicons and the scope of the significant combinations between plasmid genetic content interactions and other MGE components, which effects the dissemination of AMR genes.

## Data availability statement

The original contributions presented in the study are included in the article/Supplementary material, further inquiries can be directed to the corresponding author.

## Author contributions

SA, SF, JH, SR, YK, and DR conceived of project. SA, SF, and JH conducted the research and data extraction. SA, SF, JH, DG, and BK further analyzed the data and drafted the original manuscript. All authors contributed to the article and approved the submitted version.

## Funding

This research was supported by the National Center for Toxicological Research and U.S. Food and Drug Administration. Dr. Dereje Gudeta was supported in part by an appointment to the Research Participation Program at the National Center for Toxicological Research administered by the Oak Ridge Institute for Science and Education (ORISE) through an interagency agreement between the U.S. Department of Energy and the U.S. Food and Drug Administration. The opinions expressed in this manuscript are solely the responsibility of the authors and do not necessarily represent the official views and policy of the Food and Drug Administration or Department of Health and Human Services.

## Conflict of interest

The authors declare that the research was conducted in the absence of any commercial or financial relationships that could be construed as a potential conflict of interest.

## Publisher’s note

All claims expressed in this article are solely those of the authors and do not necessarily represent those of their affiliated organizations, or those of the publisher, the editors and the reviewers. Any product that may be evaluated in this article, or claim that may be made by its manufacturer, is not guaranteed or endorsed by the publisher.

## Footnotes

^1^ https://www.ncbi.nih.gov/pathogens/antimicrobial-resistance/AMRFinder

^2^ https://account.galaxytrakr.org

^3^ https://isfinder.biotoul.fr/

^4^ https://tncentral.ncc.unesp.br/tn_blast.html
