# Supplementary material for: In silico analyses of diversity and dissemination of antimicrobial resistance genes and mobile genetics elements, for plasmids of enteric pathogens
Source: Front Microbiol. 2023 Jan 26;13:1095128. doi: 10.3389/fmicb.2022.1095128 (PMC9908598; doi:10.3389/fmicb.2022.1095128)
Supplement: Supplementary file 1 [file Table_1.PDF]

Supplemental Table S1. Replicon typing reference sequences used in the BLAST program to detect plasmids for inclusion in the study (Carattoli et al. 2005)

IncA/C
ACGACAAACCTGAATTGCCTCCTTGATTTCGTTCAGGTTGTAACCGTGACCCATCTTCGAGA
GCTCTTTCTGGAGTTCGTACAGAGTGAACATGACTCCAGCCTTGCCCTCGATGATATGGCCTT
TCCCGTTCACTGCGAGCTTGCGTAGAGCATCCTCAAGGATTTCCTCTCGCTGTCCCGCGTAGA
TCAGCACGGTTCTTCCGTCATCCTTCTCGATGATGGCTGGCTTCACCTTCACCGTGAAATGCT
GGCCTCTGATGGTGCATTGTCGTGTCACTACAGCGTTGGATAAATCTTCATGCTCACGCTTTT
GGTCCCAAATGTACTTCGGCAGCGCATCGTAGATCTCGATGGTGTTGGAGTAGTCGTCTTTCT
TGGCCGGAGATTCAATGATCTCAAACAGGCTCATTTGCGGTGAGTCTTTGATTACGTCGAGT
TTCTCCAGGTCTTTGTCTTTGGTTCTC

IncB/O
TCTGCGTTCCGCCAAGTTCGAGGAAAAATAGTGGGGGTTTTCCTTTATGGTCTTCTGAAACTT
TTTTCAGCCTTCCGGCACCGGCAAACTGCCGTCGTTTCTGCCATACGTCCTGAATGCCTTATG
GCGCATTCTGCCGTTTTCTGGCTTTCCGGACCGC

IncFIA
CCATGCTGGTTCTAGAGAAGGTGTTGTGACAAATTGCCCTTTCAGTGTGACAAATCACCCTC
AAATGACAGTCCTGTCTGTGACAAATTGCCCTTAACCCTGTGACAAATTGCCCTCAGAAGAA
GCTGTTTTTTCACAAAGTTATCCCTGCTTATTGACTCTTTTTTATTTAGTGTGACAATCTAAAA
ACTTGTCACACTTCACATGGATCTGTCATGGCGGAAACAGCGGTTATCAATCACAAGAAACG
TAAAAATAGCCCGCGAATCGTCCAGTCAAACGACCTCACTGAGGCGGCATATAGTCTCTCCC
GGGATCAAAAACGTATGCTGTATCTGTTCGTTGACCAGATCAGAAAATCTGATGGCACCCTA
CAGGAACATGACGGTATCTGCGAGATCCATGTTGCTAAATATGCTGAAATATTCGGATTGAC
CTCTGCGGAAGCCAGTAAGGATATAC

IncFIB
GGAGTTCTGACACACGATTTTCTGTTTATTCTTTTACTGTCCACAGGCTGGAGGCTTTCTGGA
AAACGAAAATTCAGACATCAAAAAACTGTTCGGCGAGGTGGATAAGTCGTCCGGTGAGCTG
GTGACACTGACACCAAACAATAACAACACCGTACAACCTGTGGCGCTGATGCGTCTGGGCG
TTTTTGTACCGACCCTTAAATCACTGAAGAACAGTAAAAAAAATACACTGTCACGTACTGAT
GCCACGGAAGAGCTGACACGTCTTTCCCTGGCCCGTGCTGAGGGATTCGATGAGGTTGAGAT
CACCGGCCCCCGCCTGGATATGGATAACGATTTCAAGACCTGGGTGGGGATCATTCATTCCT
TTGCCCGCCATAACGTGATTGGTGACAAAGTTGAACTGCCTTTTGTTGAGTTTGCAAAACTGT
GTGGTATACCTTCAAGCCAGTCATCCCGCATGCTGCGTGAGCGCATCAGCCCTTCCCTGAAG
CGCATTGCCGGTACCGTGATCTCGTTTTCCCGTACCGATGAGAAGCACACCCGGGAATACAT
CACCCATCTTGGACAATCAGCCTACTACGATACTGAGCGGGATATTTGTTAAGTTCAGGCTTGA
TCCCCGCTGGTTGAACTGTACCAGTTTG

IncFIC
TTCTCCTCGTCGCCAAACTAGATGAAGATTATCGGGGTTTTTGCTTTTCTGGCTCCTGTAAATCCACATC
AGAACCAGTTCCCTGCCACCTTATGGCGTGGCCAGCCACAAAATTCCTTAAACGATCAGCAATCTATC

ACTCACGCCTGAGATAAGCAAGAATGTGAATATTTACAAAGTCGCTCTGCGTTTCAGCTCTGATTCAAT
CAGTTTTTCAAGCATCTGCGCCTGGGTAATACCTTCCTCATCTGCCAGTTCAC

IncFIIA
CTGTCGTAAGCTGATGGCGAAAGCCGAAGGGTTCACGTCCCGTTTTGATTTTTCCGTCCATGT
GGCGTTCGTTCGTTCGCTGGGAAAGCGTCACCGGATGCCGCCTTTGCTGCGCCGTCGTGCCA
TCGATGCGCTGCTTCAGGGGTTGTGCTTCCATTATGATCCACTGGCCAACCGTGTACAGAGA
TCCATCACCAATCTGGCTATAGAGTGCGGTCTGGCCACTGAGTCAAAAAGTGGTAATCTGTC
CATCACCCGCGCCACACGGGCGCTGAAGTTTGTGGCAGAG

IncHI1
GGAGCGATGGATTACTTCAGTACGCTTAGGCGCATTAATATAACGGGATTTGTCGTTCCCGA
TATGCTGCTCATCACTCATACATTACCCCCTTGCTTCACTGAAAAACTCTTAATCTCATTAAT
AAATTGCTGGTAGACCGCCATCACGGATTCTTCGGCAACATCAAGCTGTTTGGGAGAGCAGA
GCTCTTCTGATTTTTTGATATCCAATACGGTTCTACCCCTAGAAGCTGCGGCTTTAAACGCTT
CTGAGTTTTTGATATTTGCAGACATAAAGAGTTCCTGCACTGTCCTTACCAGTTTATCCAGGA
CAATCTTTTCATAGGGACTCTTATCGTCGACACTCACTGCCAACACTTTGAACCAGCTTAGAT
TTTCGCCCTTGCTCGGCGATTGTTTGAATCGCTCACTGATCGTCAACATGAAATCAGTTGTTG
AGGCATAATCATACTCACGAGGTGAAACGGCA

IncHI2
TTTCTCCTGAGTCACCTGTTAACACCCGGTTTCTACGCTTTACTTCATTGGCCAGTACATCCA
ACGGAAAATATCGTAACGGGGTGACAATAGCAGTGAGTTGACCCACTCACTACCATCGACA
TTCAAATCCCGGCTTCTCTGGTAGCTATAGTGACAATGGTAATGATGTGTACCTTACTGGCAT
TGTCATTGTCCTCCGAGTGTGCGTTCAGCTTTGGGAGGACACCGGAACTTAATGCGGCGAGT
CACGGCCATTGTGAATCTCGATTGGATTGGCGAATACGGCTTCCGGTTGGGCCAGATGTCAA
TGGTTATGCTTCATCCAGCTCATTACTGTAGTCATTGCCGTTCAGCTGAGCTGATGCTCCTCT
CATTCTATGTGGCGACAACGGTAGTGAGCCCAGAAAGTGACTACCGTTGTCATGGCCGTCCA
GTAGAGCGGGTCAACCTCTTAAATCACAGGGTGACAACGGTATTGAGCCCAGAAAGTCACT
ACCGATGTCATGGACGTCTGGTTAAGCGGATCAACCTCTCAATTACAGGGTGCAATGGTAGT
GAGGCCAGCAAGGCACTACCGTTGCATAGTCGTCCCGGTGNGCAGATCAACCTCTTAACTTA
CAGGATGACAACGGTAGTGAGCC

IncI1
CGAAAGCCGGACGGCAGAATGCGCCATAAGGCATTCAGGAGAGATGGCATGTACGGGCAGT
AAGTCAGAAGACTGAAGATGTTCCGGAAGCCATAAAAGGAAAACCCCCACTATCTTTCTTAC
GAACTTGGCGGAACGA

IncK
GCGGTCCGGAAAGCCAGAAAACGGCAGAATGCGCCATAAGGCATTCAGGATGTATGGCAGA
AACGACGGCAGTTTGCTGGGGCCGGAAGGCTGAAAAAAGTTTCAGAAGGCCATAAAGGAAA
ACCCCCACTATCTTTCCTCGAACTTTGGCGGGCTCGTGAAAGA

IncL/M

CTGCAGGGGCGATTCTTTAGGGGACTGGCTTTCAAGCCAGGAGATGAACTCCGGCGAAAGA
CCTTCTACTGACTCAATGTCAGAAAGCTGTAGGTATGGATTCTTTTGATTCACCAGCCCCTGC
ACATATTGGCCGGGCTGTATTGCAATAGGGTACGCATTTGCTATAATCCTTTCTGCCAGTGG
GCAGTTTGCACCCCCTGATTCTATTCCGAGCCGGCCAAAGTTCTGAAATAGGATCAGGGGGT
TTTACTTTTTGTGGCTCCTGCCACTCCTAAGCGGAACATCTCCGGGCCGCTCTTCCCGTGCTA
AATTCTGTAATAACCCCGTAAGGTTATGTAAACGCCGCTTAACATATCATGCGTTTCTAAGC
GGGGCAATACCTGCGATCCCGCAGTTACAGAGTAACCTCACTCTTCAGAATCGTCAAATTTT
AAGCCGTCGTAATAACTTTTCACAAGAAATTCAATAACCTCCGCCTGAGTGAGCTTTTTTACT
TTTGTGATCTCTATCAACATTCTTTTTGCATCTTTGGGCAGATAAAGATTTAGCTGGTCATGA
GTATCTCTGATGCGATTCCTGTAATCCTTCTGCTTATCCAGGTTACTCTTATGCACCTGCCGCT
CAAATACTCTGCTNANAAGCCTTCGACTACAACGACTNTTACCNCCTGNGTATCNCCAAAAA
ACCCGCCATANTCGGCGGGTAAACTCAAAAAATTCTGTTTATGAA

IncN
GTCTAACGAGCTTACCGAAGCTGCTTACTACCTCTCGCTAAAAGCAAAGCGCGTTCTCTGGT
TATGTCTTATGCAGACGTATTTCACAGCTTCAGTAAGCGAAGATGATGATGAGATGGCTGTA
CTCGGTGACTCTACTTTCAAAGTAAAGGTGGCTGACTATCAGCAAATTTTTCAGGTAAGCCG
TAACCAGGCTATCAAGGATGTTAAAGAAGGCGTGTTTGAGTTAAGCCGTTCTGCGGTAATCT
TTTACCCGAAAGAAGGGAGTTTTGACTGCGTCGCGCGCCCCTGGCTAACAGAGGCTGGCAGC
CGATCAGCTCGTGGTATCTGGGAAATCGAATTTAACCATAAACTCCTGCGGTACATTTACGG
CCTGACGAACCAGTTCACCACCTACTCGCTCCGCGATTGTGGCAGTCTTCGAAATCCACGGA
CGATCCGCCTTTATGAAAGTCTTGCTCAATTCAAATCTTCAGGCTTATGGGTTACTACTCATG
CTTGGTTAAATGACCGTTTCCTTTTGCCGGAATCCCAACAGAAGAACTTGGCAGAGTTGAAA
C

IncP
CTATGGCCCTGCAAACGCGCCAGAAACGCCGTCGAAGCCGTGTGCGAGACACCGCGGCCGC
CGGCGTTGTGGATACCTCGCGGAAAACTTGGCCCTCACTGACAGATGAGGGGCGGACGTTG
GCACTTGAGGGGCCGACTCACCCGGCGCGGCGTTGACAGATGAGGGGCAGGCTCGATTTCG
GCCGGCGACGTGGAGCTGGCCAGCCTCGCAAATCGGCGAAAACGCCTGATTTTACGCGAGT
TTCCCACAGATGATGTGGACAAGCCTGGGGATAAGTGCCCTGCGGTATTGACACTTGAGGGG
CGCGACTACTGACAGATGAGGGGCGCGATCCTTGACACTTGAGGGGCAGAGTGCTGACAGA
TGAGGGGCGCACCTATTGACATTTGAGGGGCTGTCCACAGGCAGAAAATCCAGCATTTGCA
AGGGTTTCCGCCCGTTTTTCGGCCACCGCTAACCTGTCTTNTAACCTGNCTTTAAACCAATAT
TTATAAACCTTGTTTTTAACCAGGGCTGCGCCCTGGCGCGTGA

IncW
GGTGCGCGGCATAGAACCGTAGGGCAGGCCGATGCTCGGCTTGCCCATGATCGACAAGGTG
ACGATGCCATTGGTGCGCTCAAAGTAGCTGGTCTTGGGGTCGGTGTGGGGCATGGTCGCTTG
CACAAGGCAACGGGCCCGTAGCCGACTAAGCCAGCTTCGCGGGCATCCTCCATTTCGAGCGC
GAGGCTCGTCTTGATGATCTCGTTGATACGATGGCCGGGGGCTTTGTTGTTCTTAGG
