# Supplementary material for: In silico analyses of diversity and dissemination of antimicrobial resistance genes and mobile genetics elements, for plasmids of enteric pathogens
Source: Front Microbiol. 2023 Jan 26;13:1095128. doi: 10.3389/fmicb.2022.1095128 (PMC9908598; doi:10.3389/fmicb.2022.1095128)
Supplement: Supplementary file 6 [file Image_1.PDF]

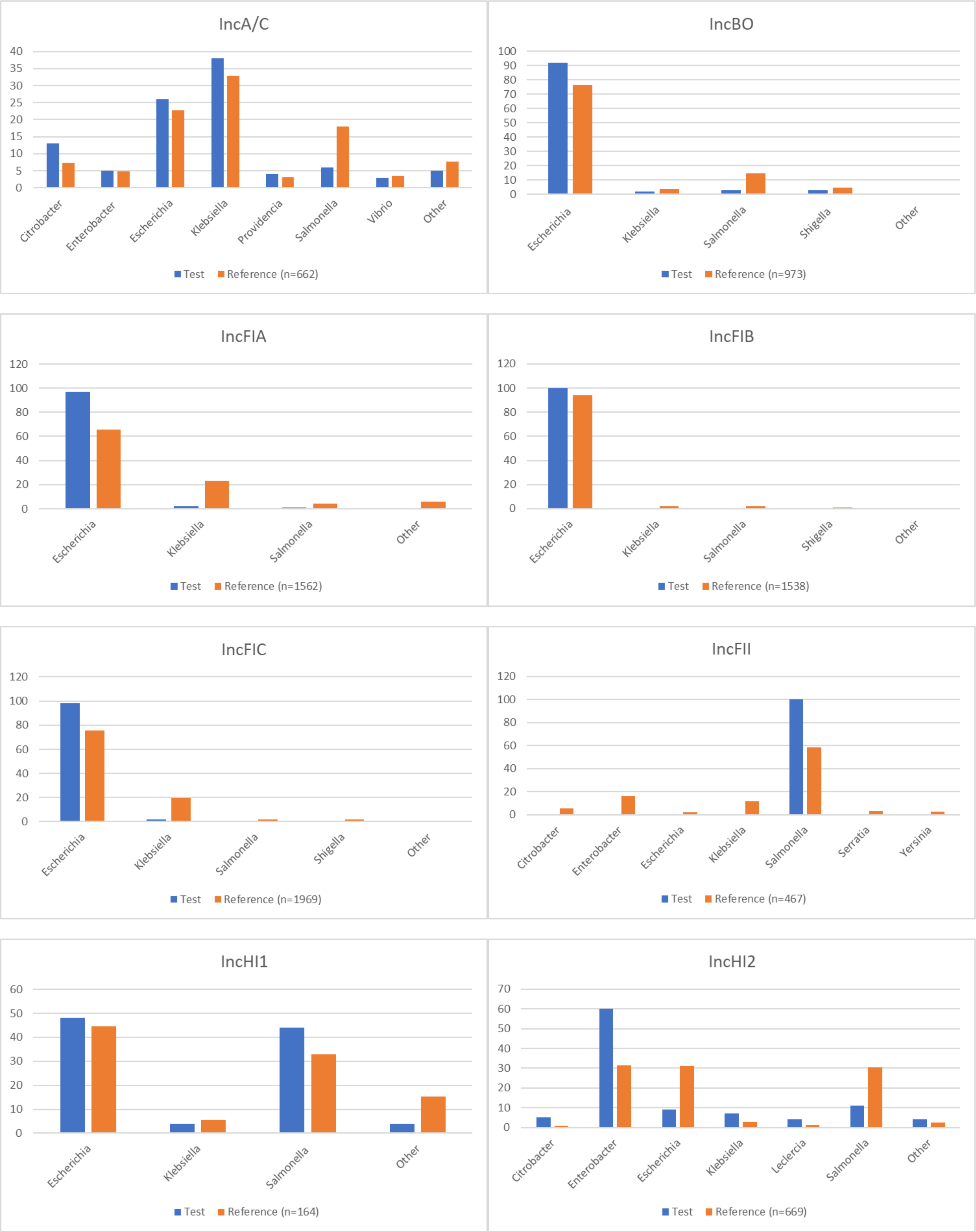
IncA/C
40
35
30
25
20
15
10
5
0
Citrobacter
Enterobacter
Escherichia
Klebsiella
Providencia
Salmonella
Vibrio
Other
Test
Reference (n=662)
IncBO
100
90
80
70
60
50
40
30
20
10
0
Escherichia
Klebsiella
Salmonella
Shigella
Other
Test
Reference (n=973)
IncFIA
120
100
80
60
40
20
0
Escherichia
Klebsiella
Salmonella
Other
Test
Reference (n=1562)
IncFIB
120
100
80
60
40
20
0
Escherichia
Klebsiella
Salmonella
Shigella
Other
Test
Reference (n=1538)
IncFIC
120
100
80
60
40
20
0
Escherichia
Klebsiella
Salmonella
Shigella
Other
Test
Reference (n=1969)
IncFII
120
100
80
60
40
20
0
Citrobacter
Enterobacter
Escherichia
Klebsiella
Salmonella
Serratia
Yersinia
Test
Reference (n=467)
IncHI1
60
50
40
30
20
10
0
Escherichia
Klebsiella
Salmonella
Other
Test
Reference (n=164)
IncHI2
70
60
50
40
30
20
10
0
Citrobacter
Enterobacter
Escherichia
Klebsiella
Leclercia
Salmonella
Other
Test
Reference (n=669)

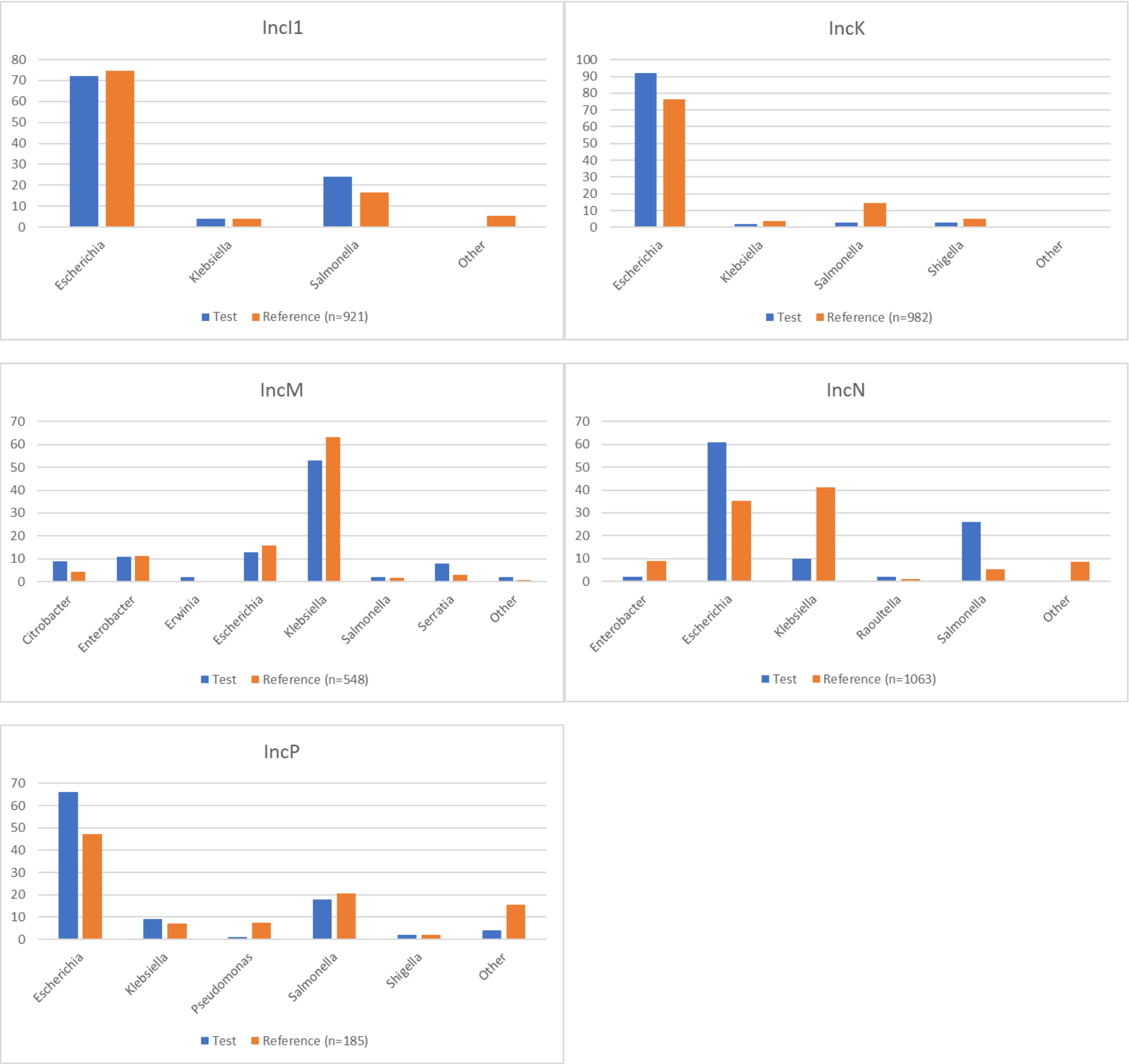


**Supplementary Figure S1.** Results of the assessment analyses to evaluate how representative the sources of the plasmids (Test) analyzed were to the larger complete plasmid data present in GenBank (Reference). The genus of the sources of the plasmids is provided along with the relative proportion of the taxa associated with the Test and Reference sets. The number of Reference plasmids for each plasmid type is shown in the graph legends. Data on IncW plasmids are not presented, since all available plasmids were analyzed in the Test set.
